# Efficacy of Gamma Irradiation in Improving the Microbial and Physical Quality Properties of Dried Chillies (*Capsicum annuum* L.): A Review

**DOI:** 10.3390/foods11010091

**Published:** 2021-12-30

**Authors:** Naleene Balakrishnan, Salma Mohamad Yusop, Irman Abdul Rahman, Eqbal Dauqan, Aminah Abdullah

**Affiliations:** 1 Department of Food Sciences, Faculty of Science and Technology, Universiti Kebangsaan Malaysia, Bangi 43600, Malaysia; spssguna@yahoo.com (N.B.); aminahsensory@gmail.com (A.A.); 2 Innovation Centre for Confectionery Technology (MANIS), Faculty of Science and Technology, Universiti Kebangsaan Malaysia, Bangi 43600, Malaysia; 3 Department of Applied Physics, Faculty of Science and Technology, Universiti Kebangsaan Malaysia, Bangi 43600, Malaysia; irman@ukm.edu.my; 4 Nuclear Technology Research Centre, Faculty of Science and Technology, Universiti Kebangsaan Malaysia, Bangi 43600, Malaysia; 5 Nutrition Department, Medicine Faculty, University of Oslo, 0372 Oslo, Norway; e.m.a.dauqan@medisin.uio.no

**Keywords:** dried chilli, gamma radiation, capsaicin, dihydrocapsaicin, aflatoxin, microbial contamination

## Abstract

Dried chilli is one of the highly traded spices globally and is well-known for its natural flavour, colour, and unique pungent taste. It is rich in nutrients and has medicinal benefits. During the dehydration and storage process, the proliferation of unwanted microorganisms in dried chilli is unavoidable. Recently, the occurrence of toxigenic fungi and faecal coliforms has been widespread that can cause severe illness and even death. Therefore, sanitation treatment is highly required to decontaminate undesirable microorganisms. Among the common sanitation treatments applied, food irradiation is gaining attention worldwide because of concern for post-harvest loss, foodborne disease, and more stringent regulation in dried chilli trading. Irradiation can successfully preserve dried chilli from pathogenic bacteria with minimal disturbance to critical physical properties, such as pungency and colour. It can also save dried chilli from secondary pollution by storing it into final packing before radiation which helps in distribution to market promptly after treatment. Furthermore, radiation does not leave any chemical residues after the treatment, ensuring the quality and safety of the dried chilli. The efficiency of radiation depends mainly on the initial level of contamination and the persistence of the harmful microorganism. A low irradiation dose is sufficient for dried chili to reduce microbial load to an acceptable level and eliminate pathogens even though a minimum radiation dose of 10 kGy is required for complete sterilization. However, high dosage may affect the colour properties. Gamma radiation, X-ray, and electron beam radiation are the three approved radiation sources for dried chilli in most countries and proven effective for dried chilli preservation. Thus, this review paper highlights the microbial and physical quality properties in gamma radiated dried chillies.

## 1. Introduction

Being one of the primary agriculture produce, spices are used throughout the world for culinary purposes. Spices can be used in various forms as it aromatizes the foods and at the same time stimulates the taste, flavour, colour, and pungency. Asian countries are the major spice consumers and now increasingly getting popularity among developed Western countries such as Europe and United States. According to a research conducted by Siruguri and Bhat (2015) [1], red chillies and turmeric were the highly consumed spices by 100% households in an urban area of Hyderabad city in India. Spices are not only known to be good for taste buds but also health purposes. For example, capsaicin in chilli helps increase body metabolism and act as an anti-inflammatory agent [2]. However, spices are mainly added on as flavouring substance for a wide range of processed foods, ready meals, and beverages. In addition, many spices are used for additional commercial purposes such as medications, perfumes, incense, soaps and condiments [3].

Developing countries are the dominant suppliers for spices in the world spice market [4]. Being grown and harvested in a hot and humid climate, proper hygienic methods must be applied for spices. Otherwise, it can lead to food spoilage and foodborne illness with high counts in bacteria, molds, yeast, and pests [5]. It is often difficult to identify food borne issues caused by spices as they are used generally in small quantities to season main dishes or meals, as focus are primarily on the main food ingredients when it comes to identifying the source. Among the spices, dried chilli is one of the largely consumed spices and the major producer in the world is India [6]. Dried chilli contamination particularly with pathogenic microorganisms, such as Salmonella causes significant public health issues all over the world. The other contaminant issue in limelight for dried chilli currently is the mycotoxin, particularly aflatoxin and ochratoxin. Both these contaminants may present naturally or accidentally in dried chilli.

Knowing the fact for health risk caused by spices, food regulatory bodies, and authorities worldwide such as the Food and Drug Administration (United States), Singapore Food Agency, and Rapid Alert System for Food and Feed (European Commission) conducts frequent inspection for the local and imported spices, particularly dried chilli and its product. For example, recently Singapore Food Agency (SFA) issued recall notice for one of the local chilli powder found with aflatoxin level exceeded the limit set by the ministry [7]. In United States, one of the famous spices and seasoning manufacturer voluntarily recalled three of the seasoning products for possible Salmonella contamination [8]. Such a product recalls are important to safeguard public health, but it causes massive damages to the environment when correct disposal procedure is not followed besides involving multiple cost factors, such as shipping, disposal, and storage. It is always good to control possible food risk problems caused by spices and spices products rather than facing all the consequences caused by them after reaching the end users. There are many methods available to decontaminate spices, particularly dried chilli and recently food radiation is gaining popularity. In this review, dried chilli radiation and its effect on the basic quality parameters will be discussed.

## 2. Food Radiation

Food radiation is one of the modern foods preserving methods, which is a kind of physical treatment that uses ionizing radiation to prevent growth or reduce the population of unwanted biological organisms [9]. The safety of the technology has been endorsed by international organizations such as the World Health Organization (WHO), the Food and Agriculture Organization (FAO) of the United Nations, the International Atomic Energy Agency (IAEA), and Codex Alimentarius. Food radiation has shown a great potential to lengthen the durability of food while maintaining its organoleptic and safety properties [10]. For the last 40 years, research has proven that radiation has enormous beneficial applications in foods, such as cereal disinfection, prevention of sprouts in potatoes and onions, maintain quality of perishable crops, sterilization of fresh and frozen meat products, seafood, and eggs. Interest in food radiation is increasing as many countries are adopting this advanced technology, and more research is being done on its potential application in food sectors. Spices are known as the greatest contaminant in foods and cause rapid spoilage if left untreated. In this context, radiation can contribute to improvement in spices wholesomeness and enhance wellbeing of people. Further, it can function as a quarantine treatment to facilitate international spices trades [11,12]. Food irradiation has shown great potential to have a longer shelf life [10]. In this treatment foods are exposed to approved sources of ionizing radiation either pre-packed or in bulk to a precisely and heedfully predetermined amounts of ionizing energy for a certain period of interval to attain the intended purposes, such as to reduce or eliminate unwanted biological colonies [9].

During food radiation, the energy of ionizing radiation passes through the food material and generates free radicals from the substance that it penetrated. Free radicals are distinctively reactive and very short-lived. However, these free radicals have restricted mobility and diffusing capability with a longer lifetime in dried and frozen foods, as well as when the food material contains hard substances such as bone. These conditions may require high levels of radiation energy to eradicate microorganisms in the food. Additionally, the efficiency of the radiation process depends on the surrounding structure of the organism and its radiation sensitivity, the degree that causes recovery of impaired DNA and most importantly the quantity of DNA present in the selected organism. So, in food radiation, the condition of food matters [13].

Radiation may cause a direct or indirect effect on chemical changes in food. In direct action, radiation directly hits the DNA molecules, disrupting the molecular structure that damages cells [14] or even causes cell death. Direct effects are typical for cells with low water content, such as dried foods. Indirect effects are caused by water radiolysis. Ionizing radiation is able to distract or disturb the structure that it binds the water molecule that produces free radicals, such as hydroxyl (•OH), hydrated electron (e^−^_aq_), hydrogenated atom (•H), and others [15]. These radicals create reaction with other food components especially foods with high moisture content, such as fresh produce, meat, and meat products, destroying the cell through indirect effect. In many foods, the changes caused by irradiation is through indirect effects [13].

When microbes present in the food are irradiated, the energy from the radiation breaks the bonds in the DNA (deoxyribonucleic acid), the largest molecules in nucleus and RNA (ribonucleic acid), causing defects in the genetic instructions. DNA consists of a very long ladder twisted into a double helix [16]. Sugar and phosphate molecules are the main components in backbone while the rungs of the ladder are comprised of four nucleotide bases (cytosine, thymine, adenine, and guanine), which are joined weakly in the middle by hydrogen bonds [17]. Breakage of these weak hydrogen bonds halts cell multiplication and eventually causes cell death while giving minimal effects on non-living tissue [18].

It matters if the food is frozen or fresh, because it takes larger radiation dose to kill microbes in frozen foods. The effectiveness of the process depends also on the organism’s sensitivity to irradiation, on the rate at which it can repair damaged DNA, and especially on the amount of DNA in the target organism [13]. Parasites and insect pests are rapidly killed with low irradiation dose due to having large amounts of DNA while bacteria requires higher irradiation dose as it contains lower amount of DNA. Viruses are generally resistant to high irradiation doses approved for foods because they are smallest in size and have nucleic acid [18]. Any foods with living cells will be damaged or killed and this is useful in extending shelf life for fresh fruits and vegetables because it inhibits sprouting and delays ripening.

### Ionizing Sources for Food Irradiation

Under the Codex Alimentarius Commission, three ionizing sources has been approved for food radiation, that are gamma-ray, electron beam (E-beam) and X-ray. Gamma rays produced by radioisotopes cobalt-60 (^60^Co) and cesium-137 (137Cs), while high energy electrons (maximum level of 10 MeV) and X-rays (maximum level of 5 MeV) are produced by machine sources [19]. These kinds of radiations are labelled ‘ionizing’ because the energy generated by them able to disintegrate molecular bonds and alter the original placement of electrons from atoms and molecules. As a consequence, two electrically charged particles (ions) are created. Electron beam sterilization is generally done with electron beams generated from accelerators which is a stream of high energy electrons pushed out of an electron gun and directed to food products. It can be simply switched on or off. These electrons able to pass through food materials with depth up to 2 to 4 inches and the treatment can be done on the top and bottom of the packages. The radiation process does not produce any radioactive materials [20].

Gamma rays and X-rays are a form of electromagnetic radiation such as radio waves, microwaves, ultraviolet, and visible light rays. In the electromagnetic spectrum, gamma rays and X-rays are in the short-wave lengths. Both are very penetrating and can pass through a food material to several feet depth. X-rays are emitted during transitions of electrons in excited atomic shells and lower in energy compared to gamma rays. Both have the same basic properties, and the key difference is how they are produced. X-rays with different ranges of energy produced by X-ray machines. To produce the X-rays, a beam of the electron is directed at gold or thin metal plate, producing a stream of X-rays. X-rays able to penetrate thick foods, in a way similar to gamma rays. Like the electron beam, the instrument can be easily operated by turning on and off. Further, it does not involve radioactive substances [21].

**Figure 1:**
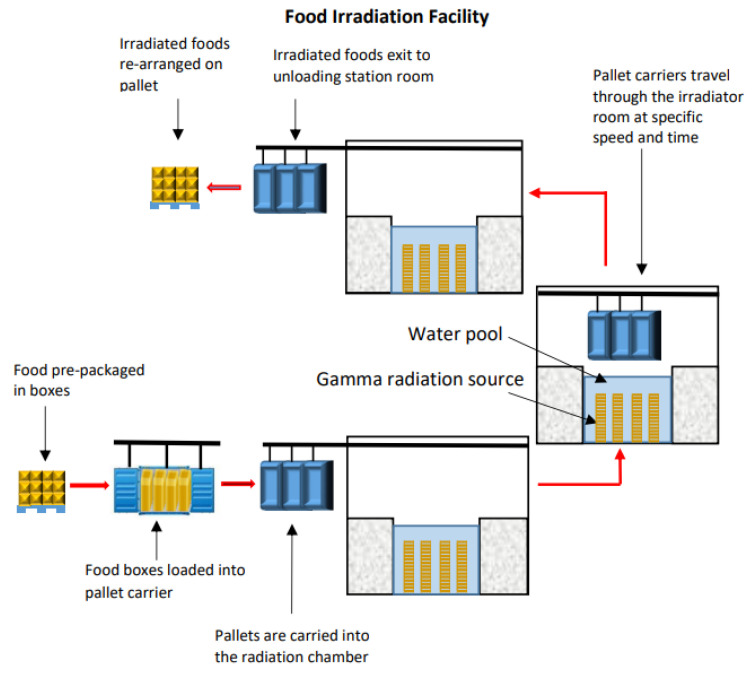
A schematic diagram of a common food irradiation facility.

Gamma rays with specific energy generally come from the spontaneous disintegration of radioactive isotopes or radioisotopes, are unstable and emits radiation as they spontaneously disintegrate or decay to a stable state. Gamma-ray is the common radioisotopes used, which is cobalt-60 (^60^Co). It is produced by neutron bombardment in a nuclear reactor of the metal cobalt-59. Radioactive substances are emitted in gamma rays all the time. The gamma-ray reactor is immersed in water filled tanks for complete protection. For the purpose of food radiation, the reactor is taken out from water and positioned in a confined area with thick concrete wall that protects the rays from released out. The materials to be radiated are conveyed into this confined wall and radiated for predetermined period of time. After the radiation process is completed, the reactor will be stored back in the water tank simply by a mechanical device [16]. Figure 1 shows a simple diagram for food irradiation facility.

## 3. Microbial Contamination in Spices

The growth of many types of microorganisms are readily supported in spices that may happen at different stages of spices processing, such as cultivation and harvesting. Thus, they are well known as a notable contaminant in foods [22]. As a common practice in developing countries, harvested spices are spread and dried on open fields, tar roads, concrete floors, or rooftops [23]. There are no treatments done to reduce the microbial load prior to selling. Improper and less hygienic post-harvest handling of spices further aggravates microorganism growth. Microorganisms generally contaminate freshly harvested spices from the environment, such as dust and animal excreta. Other possible microbial contaminations for spices are the presence of indigenous microorganisms from plants, unhygienic food processing area, air, dust, polluted water source/irrigation with the presence of human/animal excreta, improper pre- and post-harvest handling during processing, storage and distribution [24]. Consequently, this may reduce the durability of the foods added with spices uncooked or minimally processed foods that can be a great threat for health [25].

While the spices are being dried, the total microorganism colony may grow above 10^5^ per gram of material [26]. In the previous studies on bacterial profile in spices, microorganisms, such as total heterotrophs, *Bacillus cereus*, *Clostridium perfringens*, *Escherichia coli*, *salmonella,* and toxigenic molds were among the microorganisms detected in spices [27]. Studies showed packed spices had lower contamination compared to unpacked. [26], evaluated microbial quality for nine types of spices from Tehran retail market for packed and unpacked samples. The study revealed 63.2% of the samples had more than 5 × 10^5^ cfu/g aerobic mesophilic bacteria, 24.8% of the samples had coliform count above 10^3^ MPN/g, 21.9% of the samples had more than 5 × 10^3^ cfu/g mold count, and 23.4% of the total samples were detected with *E. coli*. In comparison to packed spices, the non-packed spices had higher coliform and *E. coli* counts. In a related study [24] observed unpacked spices had plate count up to 10^5^, whilst the packed one was 10^3^. Interestingly, in this study coliform and *Escherichia Coli* were not found in packed red pepper but were reported in unpacked.

When spices are used as a seasoning in processed food without any sterilization step the initial count of the microorganisms may spoil the food at a much faster rate. This will eventually lead to severe food poisoning, depending on the species and contamination level in the prepared food. Any sterilization technique that involves moisture, heat, or steam treatment is not an ideal technique for dried spices because spices are very sensitive to water and temperature, which may degrade the colour and reduce the flavour [28]. Previously, it was a common practice for spice dealers to use ethylene oxide (ETO) for disinfection till it was declared as carcinogenic and banned by few countries [29]. Thus, radiation is considered as a promising technique to reduce or completely sterilise spices [30]. For commercial purposes, spices radiation has been permitted and implemented internationally for years. The Codex Alimentarius has set 10 kGy as the maximum allowable dose for food radiation. This is similar to the standard applied by Malaysia and United Kingdom. Under the Australia New Zealand Food Standards Code, herbs and spices may be irradiated to control germination and insect activities, as well as to manage weeds. For this the exposure of irradiation dose must be lower than 6 kGy. For bacterial decontamination purposes, the radiation dose approved is between 2 and 30 kGy [31]. Japan’s standards for food radiation are the most stringent in the world. Japan Food Sanitation Law enforced in 1947 prohibits the sale and processing of radiated spices [32]. In the United States, the Food and Drug Administration authorized less than 30.0 kGy radiation dose for spices.

Gamma irradiation between 5.0 to 10.0 kGy generally meets expectation in eliminating microbial contamination and insects without altering chemical or taste and flavour profile, depending on the variety of the spices [28,33]. However, the radiation dose required to decontaminate relies mainly on initial microbial load and the type of microorganism present in the spices [30]. In general, many researchers reported that fungi and coliforms can be killed with much lesser radiation dose in comparison to those needed for total bioburden [34].

## 4. Quality Parameters in Radiated Dried Chilli

In Malaysia, one of the highly imported spice is dried chilli (*Capsicum annuum* L.) and the primary source is from India [6]. Dry chilli is the major ingredient used by Malaysians to make authentic local spicy dishes like ‘sambal’, curry, and other exotic dishes, such as ‘satay’ sauce, ‘rendang’, and ‘asam pedas’. In addition, spiciness, dried chilli also adds value to the dish prepared with its bright red natural colour and flavour [35].

The radiation dose required to decontaminate dried chilli relies on the initial level of contamination and type of the microorganism present. Generally molds, fungi, and coliforms are eliminated by doses lower than those required for total bacteria [34]. A minimum dose of 4 to 5 kGy is able to eliminate fungi and coliforms while some of the bacteria and yeast achieves complete sterilization with minimum 10 kGy radiation dose. Controlled or low level of gamma irradiation for dried chilli can kill microorganism effectively without affecting colour, flavour, and functional properties because gamma irradiation is known as cold sterilization as it only introduces little to no amount of heat to the food material [36].

**Figure 2:**
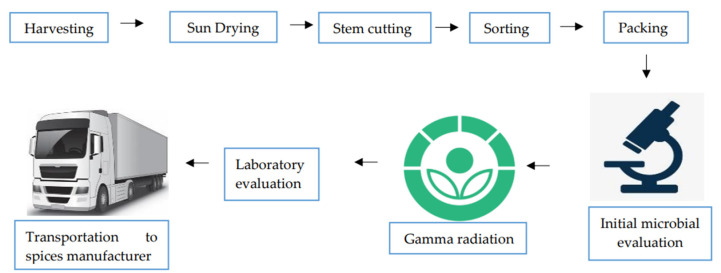
Diagram for dried chilli radiation process from harvest.

Undoubtedly, radiation treatment is very efficient against bacteria, in comparison to any other treatment that produces heat. Further, radiated materials are free from chemical residues [11]. As for dried chilli, it can be radiated with gamma rays emitted from radioisotopes ^60^Co and ^137^Cs or high energy electron and X-rays produced by machine sources. Jung et al. (2015) [37] evaluated the effect of X-ray, gamma-ray, and electron beam irradiation (2 to 10 kGy) for total aerobic microbes (TAM), capsaicinoids and capsanthin, colour, and sensory properties of red pepper powder. The author observed at 6 kGy, all the radiation sources can reduce the total aerobic population efficiently without compromising to quality parameters evaluated. However, many sensory panellists detected an off flavour for all the irradiated samples. Finally, the author remarked X-ray can be used to irradiate dried spices in the same way as gamma rays and electron beam does. Figure 2 shows an example diagram for dried chilli radiation from farm to spices industry. Incorporation of radiation treatment is currently being practiced after the dried chilli is packed in suitable size and initial quality is pre-determined.

### 4.1. Effect of Gamma Radiation on Microbial Quality in Dried Chilli

Heavy microbial contamination in dried chilli have been widely reported in many research works. Ikuomola and Eniola (2015) [38] evaluated the microbial profile for sun-dried pepper (*Capsicum annuum*) from open markets in Nigeria. The researcher observed high total viable counts, coliform and fungal counts in between 6.30 to 6.65, 6.00 to 6.48, and 5.30 to 6.30 log10 cfu/g, respectively. A total of 15 bacterial species were isolated in their findings. In another study, Erdem et al. (2013) [39] examined possible pathogens, namely *Salmonella* spp. And *Aeromonas* genus in 40 unpackaged and 10 packaged red pepper samples collected from retail shops in Istanbul. *Salmonella* spp. Was absent in all the samples, while 10% from the samples were detected for *Aeromonas* spp. The observation revealed contamination in unpackaged red pepper samples is higher than that of packaged samples. Zaini et al. (2010) [40] reported high mold count in dried chilli, thus the evaluation on mold and aflatoxins are also prioritized in dried chilli.

Even though many researchers evaluated effect of gamma radiation different quality parameters, such as pungency, sensory, microbial content, carotenoids, and ASTA colour value in dried chilli, microbial quality is given utmost attention. Odai et al. (2019) [41] evaluated decontamination in Legon-18 pepper powder using gamma radiation in their research work. Sterility test was conducted for the samples radiated at 1, 2, 4, and 5 kGy, in which the test materials were artificially contaminated with predetermined number of colonies of *E. coli*, *Listeria monocytogenes* and *Salmonella enterica* Typhimurium. The inoculated pepper powders were sterilized at 5 kGy. In a related study by Deng et al. (2015) [42], dried chilli (*Capsicum frutescens*) and Sichuan pepper (*Zanthoxylum bungeanum*) were irradiated with gamma-ray after artificially inoculated with *E. coli*, *Salmonella enterica* Typhimurium and *Aspergillus niger*. The author concluded 4 and 5 kGy radiation doses are suitable to eliminate almost all the microorganisms tested in both the spices. *Aspergillus niger* was not detected at 1 and 1.5 kGy radiation for dried chilli and Sichuan pepper, respectively.

Rico et al. (2010) [28] studied steaming and gamma irradiation effect on microbial properties for dried red pepper stored at refrigerated and room temperature, 4 ± 2 °C and 20 ± 2 °C, respectively, for 6 months. From the initial microbial load of 10^6^ cfu/g, radiation successfully reduced by 5 log in comparison to steam treatment which reduced at much lower rate by 1 to 2 log. In this study, it was recommended that irradiation together with refrigerated storage will minimize the changes in physicochemical properties in dried red pepper.

Iqbal et al. (2013) [10] evaluated gamma irradiation and its effect on microbial contamination for chilli samples obtained at various Punjab districts in Pakistan. The authors observed radiation at 6 kGy decreased the fungal contamination by 5 log. Similarly, Iqbal et al. (2012) [43] found radiation treatment at 2 and 4 kGy reduced fungal contamination by 1 and 2 log, respectively, for hot peppers. However, mold growth was not observed immediately after radiation at 6 kGy and after storage for 3 months. This shows mold becomes an easy target at 6 kGy gamma radiation.

In a related study, Yu et al. (2017) [44] investigated radiation impact on microbial quality and aflatoxin B1 in ground red pepper powder collected locally. The samples were vacuum-packed in aluminium foil bags and radiated at 0, 3, 6, 9, 12, 15 and 18 kGy. Storage evaluation was done for 8 months at 4 °C. The results revealed that 18 kGy dose effectively decontaminates red pepper powder, with a 6-log reduction in total aerobic bacteria count. The mold count decreased as well but did not show any significant effect on aflatoxin B1.

Helga et al. (2018) [45] from Hungary evaluated gamma irradiation effect, steam treatment, microwave heating, enhanced microwave treatment, and radio-frequency heat treatment on biological quality, bioactive component, and organoleptic properties for ground dried paprika. Gamma radiation treatment was done at 1, 5, and 10 kGy. The author observed radiation treatment was highly effective against reducing and eliminating microbial count in comparison to other treatments. Similarly, Kitai and Furuta (2009) [46] in their research reported 10 kGy irradiation for paprika powder was enough to attain sanitation level enforced in Japanese Food Sanitation Law.

Song et al. (2014) [47] studied eliminating food pathogenic bacteria in dried capsicum by means of gamma radiation and in this study the samples were artificially contaminated with *Escherichia coli O157: H7* and *Salmonella Typhimurium*. The inoculated samples were radiated at 0, 1, 2, 3, and 5 kGy. The author observed inverse effect to the strength of radiation dose versus number of pathogenic bacterial colonies survived. It was concluded that gamma radiation could be a good source of non-thermal treatment to inactivate pathogenic bacteria in spices with minimal colour change.

### 4.2. Effect of Gamma Radiation on Pungency (Capsaicin and Dihydrocapsaicin) in Dried Chilli

Pungency is a critical quality criterion in chilli industry. Pungency in dried chilli is derived from capsaicinoid compounds containing amide acids from vanilinamine and fatty acid chains branched at C9 and C11. The pungent components are produced in the glands and in the inner white pith or rib of the chilli. The white rib is the part that runs down the middle and alongside the chilli and holds the chilli seed. These are the hottest parts of the chilli as capsaicinoids are concentrated in these parts. Chilli seeds are often hot because they are attached to the white ribs [48]. The pungency value is rated by Scoville Heat Unit (SHU). The measurement unit was termed following its invention by an American Pharmacist, Wilbur Scoville, in 1912 and the testing method was known as Scoville Organoleptic Tests [49]. Since then, many other methods were created and evaluated for pungency components measurement in chillies. Among those, analysis using High Performance Liquid Chromatography (HPLC) is well accepted for its accuracy and reliability which than replaced the organoleptic method [50].

**Figure 3:**
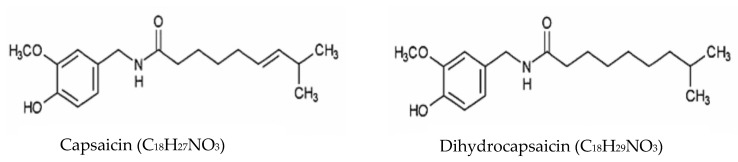
Chemical structure of capsaicin and dihydrocapsaicin [54]. Copyright: Journal Cancer Letters, 2001.

Figure 3 shows the chemical structure of capsaicin and dihydrocapsaicin. These two components contribute to approximately 90% of capsaicinoids in chilli fruit [51]. The Chemical Abstract Service (CAS) number for capsaicin is 404-86-4 with molecular formula C_18_H_27_NO_3,_ and the molecular weight is 305.42. Dihydrocapsaicin CAS number is 19408-84-5 with molecular formula and molecular weight are C_18_H_29_NO_3_ and 307.43, respectively [52]. Capsaicin and dihydrocapsaicin can cause severe skin and eye irritations thus, these two compounds play a vital role in pepper spray manufacturing used for self-defence. However, it also has several medicinal uses such as pain killers for sore muscle, skin irritants, arthritis, and anti-inflammatory agents (Mukund et al., 2014) [53].

Limited studies are available on gamma radiation and its effect on capsaicin and dihydrocapsaicin level in dried chilli as mostly importance was given for microbial profile. Ref [55] evaluated capsaicin and dihydrocapsaicin content for sun-dried hot paprika pre-packed in synthetic low-density polyethylene bags and radiated at 2, 4, and 6 kGy. The radiated and control sample pack were kept and evaluated for 90 days at 25 °C. Immediately after radiation the capsaicin and dihydrocapsaicin levels remained stable at 2, 4, and 6 kGy. However, at the end of the storage period, capsaicin showed an average reduction of 3.4%, while dihydrocapsaicin was 4.2%. The author observed the analysed capsaicinoids were more stable at 6 kGy at the end of storage for 6 kGy.

In a related study Odai et al. (2019) [41] evaluated effect of 5 kGy radiation on capsaicin, dihydrocapsaicin and total capsaicinoids for Legon 18 pepper powder. The author found pungency value increased after radiation which could be attributed by induction of water deficit by gamma radiation. However, the author observed a decrease in pungency value with an increase in storage time resulting from milling effect and residue enzymatic reaction. This is in good agreement with findings reported by Yu et al. (2017) [44] and Giuffrida et al. (2014) [56].

### 4.3. Effect of Radiation on Aflatoxin B1, B2, G1, and G2 in Dried Chilli

**Figure 4:**
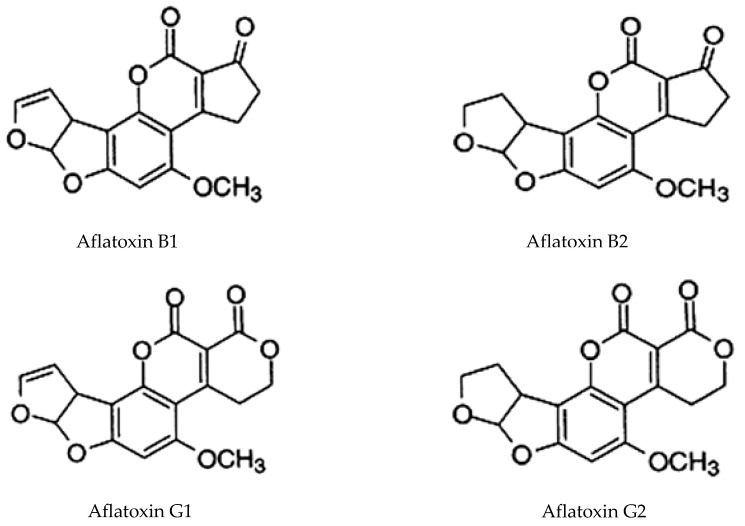
Chemical structure of aflatoxin B1, B2, G1, and G2 [59]. Copyright: African Journal of Food Science, 2010.

Aflatoxins have greatest influence in determining dried chilli quality because it degrades the dried chilli quality and render it unfit for consumption. Aflatoxins are highly toxic and well known as cancer causing substance, produced by some strains of *Aspergillus flavus*, *Aspergillus parasiticus,* and *Aspergillus nomius* molds as by produtcs [57]. *Aspergillus flavus* produces aflatoxin B1 and B2 while *Aspergillus parasiticus* produces aflatoxin G1 and G2. Figure 4 shows the chemical structure of aflatoxin B1, B2, G1, and G2. Aflatoxins are commonly detected in dried chillies globally. If aflatoxin contamination has occurred, it is impossible to destroy them by cooking with temperatures up to 100 °C because they are very stable against chemical and temperature. Although higher food preparation temperatures, such as in roasting, frying, toasting, and extrusion, may show reduced risk in aflatoxin contamination [58].

Global standards and guidelines have been developed to ensure consumers are well protected from foods and commodities known to be contaminated by aflatoxins. For spices including *Capsicum* spp. The European Commission has restricted to 5 μg kg^−1^ as the permissible level for aflatoxin B1, while for the total aflatoxins it should be less than 10 μg kg^−1^ [60]. Malaysian government has set a maximum limit of 5 μg kg^−1^ for general foods including dried chilli [61]. Literature have shown aflatoxin contaminations in dried chilli have exceeded the limit set in EU and local food regulation [62,63]. It is essential to understand that although aflatoxins are often contaminated in primary foods by mold growth that can be eliminated easily, the toxins produced are very stable and can resist severe processes [64]. This could be a potential thread in ready to eat food and ingredients, such as chilli paste, peanut butter, and roasted coffee powder.

High-level contaminations in dried chilli are commonly found in warmer parts of the world with higher climate change or variation [65]. Aflatoxin contamination in dried chillies are higher than the regulated level, probably be due to producing country’s hot and humid climate, violation in sanitation practices during harvesting and processing, mold development, and inappropriate storage conditions [66]. However, aflatoxin contamination was also proven to occur in cold-room stored dried chillies [67]. Suitable analytical procedures meant for aflatoxins identification and evaluation is crucial to compliance with existing regulatory levels and ensure dried chilli is safe for consumption and trade. Many developing countries with aflatoxin issues find it difficult to screen mycotoxins in foods due to cost factor and lack of experts to maintain analytical laboratories [68].

Numerous incidents were reported about aflatoxin in dried chilli and this really affected the global trade, beside causing a lot of wastages. Gamma radiation is an advanced procedure, believed to contribute to aflatoxin reduction in food and feed. It is a known fact that low radiation dose is able to eliminate mold growth and eventually reduces the risk for aflatoxin production, but a higher radiation dose is required to destroy aflatoxin produced especially in low moisture products, such as dried chilli. This was explained by Di Stefano and Pitonzo (2014) [69] in their study that radiolysis of water happens during gamma radiation and free radicals are produced that could act on double bonds especially on heterocyclic rings of mycotoxin, creating an energetically positive reaction being reason for aflatoxin destruction to a lower level in their study material. Generally, the reaction of gamma radiation to aflatoxin can be observed in two ways: directly acting on aflatoxin producing molds under specific conditions to eliminate toxin production, and indirect contribution to a reduction in aflatoxin production by eradicating mold survival which leads to the avoidance of aflatoxin producing fungal growth.

There are limited literature available about effect of radiation on aflatoxin contaminated dried chillies. However, the effect of irradiation on molds especially on *Aspergillus flavus* and *Aspergillus parasiticus* are satisfactorily available. Some of the available literature claimed reduction in aflatoxin as an impact from gamma radiation in dried chillies. Nevertheless, contradicting results were also observed in some studies. In a related study by Muhammad et al. (2009) [70], aflatoxin-contaminated red chilli samples were packed in HDPE bags and gamma-irradiated at 2, 4, and 6 kGy. Storage evaluation was done at room temperature. As an impact of radiation, aflatoxin level decreased from 3.35 µg/kg to 3 µg/kg and upon completion of storage period a reduction of 11% in average was achieved with an increase in radiation dose up to 6 kGy. The author concluded that chillies radiated after being packed into high-density polyethylene (HDPE) bags will eliminate the possibilities of re-contamination and can be safely stored for better quality retention. It was also recommended to irradiate dried chillies at higher irradiation dose to see the effectiveness against complete aflatoxin eradication.

In another study, hot peppers tainted by molds and aflatoxins during processing were collected and packed in polyethylene bags and radiated at 0, 2, 4, and 6 kGy. The results indicated that mold is susceptible to gamma radiation at 6 kGy and zero mold growth was observed even after 3 months of storage. However, total aflatoxins were not effectively reduced after radiation [43]. In a related study, the gamma irradiation and its impact on fungal load and aflatoxin reduction in the ground and whole chillies were evaluated. The initial isolation of *Aspergillus* species from the toxin-producing molds within samples collected showed *Aspergillus parasiticus* were predominant. The isolated fungi and the chilli samples collected were exposed to gamma radiation at 2, 4, and 6 kGy and evaluated for aflatoxin B1 and total aflatoxins. For the ground chilli samples, a maximum reduction of 98% was observed for aflatoxin B1 from its initial value, 20 µg/kg while for total aflatoxins, 91% of the reduction was achieved from the initial value of 22 µg/kg. As for the whole chilli, 97% and 92% reduction were achieved for aflatoxin B1 and total aflatoxin, respectively. It was concluded aflatoxin contamination decreased as the radiation dose was increased, even though complete removal for aflatoxin was not attained [10]. Byun et al. (2019) [71] evaluated the effect of gamma-ray, electron beam, and X-ray on of *Aspergillus flavus* reduction in ground red pepper and gochujang (paste produced from red pepper). *Aspergillus flavus* was effectively reduced by 4 logs upon radiation with gamma-ray and electron beam at 3.5 kGy any impact to physicochemical quality. The researcher concluded that gamma irradiation effectively controls aflatoxin-producing fungi in the samples tested.

Other than chilli and chilli related products, many researchers investigated the effectiveness of gamma irradiation on aflatoxins for foods and spices. As an example Jalili et al. (2012) [72] found that a radiation exposure of less than 10 kGy had no effect on aflatoxins reduction in black and white pepper. The maximum reduction was observed at 30 kGy. Ghanem et al. (2008) [73] reported positive correlation for aflatoxin B1 degradation with an increase in irradiation dose for food crops (peanut, shelled pistachio, unshelled pistachio, rice, corn) and feed crops (barley, corn, and bran) with radiation at 4, 6, and 10 kGy. Even though complete destruction for aflatoxin B1 was a failure, maximum degradation of 80% was observed for corn at 10 kGy. In a related study, rice samples were radiated at 0, 2, 4, and 6 kGy. The highest aflatoxin degradation with more than 95% was observed at 6 kGy radiation dose [74].

### 4.4. Effect of Gamma Radiation on Moisture and Water Activity (aw) in Dried Chilli

Moisture or water content of food is the total available water in the food. The processing manner determines their final moisture content. Water activity is the quantity of unbound water in a sample. Notably, it influences the organoleptic properties of food, such as texture, odour, taste and colour, and shelf life in terms of resistance to microbial attack, and enzymatic and non-enzymatic reactions during processing and storage [75]. Moisture content and water activity are correlated to dried chilli shelf life and its final quality. High moisture content and water activity can cause fungal growth, and thus introduces a high risk of aflatoxin production. On the other hand, low moisture content can accelerate pigment degradation and colour loss [76] and browning compound development [77]. It is important to dry the chilli to a suitable level of moisture content.

Manjula and Ramachandra (2014) [76] studied the effect of drying method on the physical and chemical characteristics of Byadgi variety chilli. They observed 10% (d.b.) average moisture content in dried chilli and the water activity varied between 0.51 to 0.68 *aw*. The authors highlighted Indian dried chillies have a moisture content of around 16% (d.b.) and for the export purposes, moisture content is limited between 10% to 11%. Mona et al. (2014) [78] studied different drying techniques and evaluated moisture content in Serrano and Fresno type chillies. Before drying, the moisture content was reported as 84.3 and 86.06% and after drying the value decreased to 7.2 and 8.46% for Serrano and Frenso chillies, respectively.

Toontom et al. (2012) [79] observed in their study that fresh chillies had an initial moisture content of 85.15% and water activity of 0.99 *aw*. After drying, the average moisture content was 11% (wb) and the water activity ranged from 0.51 and 0.68 *aw*. Zaki et al. (2013) [80] evaluated moisture content in Moroccan paprika at different intervals in a year. Moisture varied between 8.58% and 10.8%, with no discernible difference in the samples taken. The author suggested dried paprika to have a moisture content below 11.0% to inhibit mold growth. Furthermore, the author implicated a strong relationship between high moisture and less stability of ascorbic acid and colour pigments. Venkata et al. (2014) [81] in their review mentioned that moisture content in dried chilli should be reduced to 10% from its initial value around 65% to 80%.

Generally, lower moisture content and lower water activity contributes to storage stability for dried chilli. However, these good attributes cause much weaker lethal destruction for microorganisms ionizing radiation because much lower free radicals are generated [43]. In contrast to other quality indicators in dried chillies, such as microbial load, colour, nutritional value, and spiciness, the effect of radiation on moisture and water activity in dried chillies has not been substantially researched.

Muhammad et al. (2009) [70] investigated the impact of radiation on aflatoxin contaminated chillies obtained at a local market. The samples were radiated with cobalt 60-gamma irradiator at 2, 4, and 6 kGy. The irradiated and control samples were subjected for storage evaluation at ambient condition for 90 days with relative humidity of 45–60%. The initial moisture content for the control sample and after radiation at 6 kGy was 11.41 and 11.39%. Overall, the sample moisture content interacted insignificantly to radiation doses applied and storage up to 90 days.

In another research, the effect of steaming and irradiation treatment (10 kGy) on red pepper powder was evaluated and compared at ambient (20 ± 2 °C) and refrigerated (4 ± 2 °C) storage condition by Rico et al. (2010) [28]. After radiation at 10 kGy, the moisture content reduced to 9.04% as compared to control sample with 10%. The moisture content of the samples kept at room temperature reduced even further and more remarkable after six months storage, to 7.75% for control, 8.47% for steamed and 8.74% for radiated red pepper powder. According to the author, radiation can cause depolymerization of polysaccharides, which can cause significant changes in the cell membrane and connective tissues, resulting in softening and easier water release in radiated foods.

In a related study by Iqbal et al. (2012) [43], dried hot peppers were packed and irradiated at 2, 4, and 6 kGy in Co^60^ gamma irradiator. The radiated samples were stored with a control sample at 25 °C for 90 days and evaluated for mycological, aflatoxins, and moisture. The initial moisture content for control sample varied between 11.96% and 12.52% and immediately after radiation at 6 kGy, the value decreased to 11.87–12.46%. The researcher observed an average 1% moisture loss in radiated samples and upon storage, the moisture content did not change significantly.

Jalili et al. (2012) [72] briefly explained the important effect of water in destroying aflatoxin and ochratoxin by gamma irradiation. The primary reaction of radiation in food is the ionization of water, which disassociates the water molecules through radiolysis. This results in a positively charged radical ion and a free electron that moves quickly. Through dipolar interactions, the electron is grabbed by water and becomes solvated. This is referred as an aquatic electron or a solvated electron, or it can react with H+ to generate radical. The water radical ion can split into a hydroxyl radical and a hydrogen ion when it dissociates. In the end, hydrogenated electrons, hydroxyl radicals and hydrogen ion and hydrogen atoms are produced. These components are expected to interact with double bonds of aromatic or heterocyclic rings in aflatoxin B1 and G1 molecules and induce chemical changes by causing its degradation or remodelling.

### 4.5. Effect of Gamma Radiation on Surface Colour (L*—Lightness, a*—Redness, b*—Yellowness) in Dried Chilli

Two important criteria that determine the quality of dried red chillies are its natural red colour and pungency. These two directly influences the quality and commercial value of the food when it becomes part of the ingredient [82]. Colour parameter for dried chilli is crucial because consumers conclude its colouring power based on the visual appearance, which influences customer selection [83]. Apart from that, surface colour may tell us about the properties of the food itself, such as if it is good enough to be used or suitable for the intended purpose [84].

Chilli consists of numerous unique carotenoid pigments that are developed during ripening and are accountable for the rich and deep red colour. The essential carotenoid pigments are capsanthin, capsorubin, and cryptocapsin that give the chilli intense red colour. Although yellow orange colour is responsible for beta-carotene, zeaxanthin, violaxanthin, and beta-cryptoxanthin. Most of the chilli varieties consist of 30–70% of carotenoids contributed by capsanthin [85]. Therefore, dried chili has high chances of losing its colour due to heavy microbial contamination and improper storage condition [86]. For example, mold growth may develop yellow and black spots on dried chilli surface. Storage at low temperature may help inactivate microbial growth and colour degradation, but problems may occur once used in other foods, such as reduced shelf life. Dried chilli and its derivatives also change to a darker shade due to enzymatic browning reaction during storage, affecting the dried chilli quality [83].

Minimal literature reviews are available for assessment of gamma radiation and its effectiveness on L*, a*, b* surface colour for dried chillies. Extractable colour values (ASTA) and carotenoid content were given more importance in dried chilli colour evaluation. In one of the research, Helga et al. (2018) [45] evaluated effect of radiation on carotenoid content and ASTA colour value for spice paprika at 1, 5, and 10 kGy dose. At 10 kGy the total carotenoids content and ASTA value decreased from its initial value of 2555.6 to 952.5 µg/g and 106.4 to 37.6, respectively. The researcher concluded ionizing radiation decreases total carotenoid and ASTA colour value in paprika. Iqbal et al. (2016) [55] observed decreased carotenoid value in hot paprika sample decreased after radiation at 2, 4, and 6 kGy, respectively. After 3 months of storage, an average 12% carotenoid loss was observed for radiated and non-radiated hot paprika samples. The author highlighted carotenoids are sensitive to gamma irradiation and the decrease could have been caused by an increase in the oxidation reaction which is due to energy absorption during irradiation. Opposingly, Yu et al. (2017) [44] did not identify any significant effect on red pepper powder colour upon radiation at 18 kGy.

In another study [28], Hunterlab colour values for lightness (*L*), redness (*a*), yellowness (*b*) were evaluated for steamed and gamma radiated ground red pepper. The radiation dose tested was 10 kGy. The samples were stored at 4 ± 2 °C and 20 ± 2 °C up to 6 months. Immediately after radiation, the red pepper samples showed better colour properties compared to steamed and control samples with *L* value slightly decreased to 50.10 from 50.85 while *a* value slightly increased to 20.78 from 21.53. However, *b* value did not show any difference between control and irradiated samples. After 6 months of storage at 4 ± 2 °C and 20 ± 2 °C, irradiated samples showed higher *L*, *a,* and *b*-values than the steamed sample. Overall, it was concluded that radiation at 10 kGy showed better colour properties in red pepper powder as compared to steam treatment. The author recommended refrigerated storage conditions for radiated paprika to minimize changes in physicochemical properties.

In a related study, Pouya et al. (2016) [87] evaluated and compared the effect of steam-microwave and gamma irradiation at 5 kGy on physicochemical changes in red pepper. Surface colour properties were evaluated by colorimetric measurement with Hunterlab system for L*, a*, and b*. In this study, radiation at 5 kGy showed better colour preservation in contrast to the control and steam-microwaved samples. Radiated red pepper showed highest lightness (L*), redness (a*), and yellowness (b*) values. Overall, the author observed better colour preservation in red pepper powder radiated at 5 kGy, while control and steam-microwaved samples showed colour loss.

### 4.6. Effect of Gamma Radiation on Other Quality Parameters in Dried Chilli

Studies have shown that irradiation treatment minimally affects the quality parameters in dried chilli such as total carotenoids, volatile compounds, vitamins, ascorbic acids, and phenolic compounds provided appropriate radiation dose is applied. Radiation effect especially at low and medium radiation doses on vitamins are not really considerable, being reason vitamins are comprises of smaller molecules and not largely found in foods. Further, vitamins are vulnerable to any kind of food treatments, particularly water soluble vitamins. Fat soluble vitamins are known to be more sensitive to irradiation treatment [88].

In one of the study by Iqbal et al. (2016) [55], radiation dose up to 6 kGy did not affect the initial level of ascorbic acid and total phenolics even though a slight drop in carotenoids was observed. Similarly Muhammad et al. (2009) [70] in his study demonstrated insignificant changes in phenolics compound and stable proximate content in dried red chillies after radiation at 2, 4, and 6 kGy. In a related study Kispéter et al. (2003) [89] concluded that ionizing radiation is a better choice in comparison to steam treatment in preserving dried paprika quality for colour, viscosity, and free radical content.

Rahman et al. (2021) [90] evaluated sensory properties for radiated and non radiated red chilli. The scores from the sensory panellists showed that both radiated and non radiated samples had insignificant difference and were highly acceptable in terms of colour, texture, and flavour. Similar findings were also reported by Jung et al. (2015) [37]. The researcher reported the sensory panellist easily recognised off-flavour for irradiated paprika powder samples. In a related study comparative sensory study was conducted for steamed and radiated red pepper. The researcher observed sensory scores odour was slightly affected for radiated red pepper [28].

## 5. Consumer Perception about Food Radiation

The most significant challenge for food radiation development is the consumer’s traditional belief that the radiated food becomes radioactive and causes serious illness. Fear about upgrading poor quality food materials using this high technology also exist among some of the consumers. Further, the misconception about excessive nutrient loss in radiated food is another set back in food radiation progress. Consumers should be aware that all the food processing or preservation methods causes certain impact to the natural properties of the foods. For example, food preservation that uses heat denatures vitamins and nutrients to certain level, which depends on the type of technique applied. However, radiation does not introduces heat and one can expect for better nutrient retention than any other processes that involves heat. Apart from these, consumer beliefs that radiated foods only benefits the industries and not the end users because radiated foods capable of staying longer on the shelf and consumers may really not get the fresh foods as expected. Education and awareness plays a very important role in convincing consumers about the advantages of radiated foods. Consumers are likely to accept radiated foods if proper information about food radiation is provided. Many surveys worldwide showed a positive response from consumers about the acceptance of radiated foods [11,16,91].

## 6. Conclusions

Dried chillies have bad microbial profile due to its nature of production. By radiating dried chilli, there are high chances to control the presence of foodborne pathogens besides reducing microbial load, increasing toxicological safety, and extending the shelf life with minimal changes to its physical qualities, such as moisture, pungency, and colour. This will definitely improve international trading. Generally, more than 10 kGy radiation dose is required to achieve complete sterilization in dried chilli. However, initial microbial evaluation is important before deciding the ideal radiation dose. At lower irradiation dose fungi spores may survive, thus proper storage is required to minimize the development of active propagules. Pungency in dried chilli is not affected by gamma irradiation. However, pungency may drop in radiated dried chilli if storage condition and storage time is not taken care well. Surface colour properties in dried chillies are not affected by low gamma irradiation dose. Decontaminating viable mold spores by gamma irradiation before aflatoxin production is the most acceptable method to preserve dried chilli from aflatoxin contamination. Despite this, dried chilli radiation should be practiced concurrently with stringent sanitation practices with good handling and storage procedures to avoid further microbial multiplication and deterioration in quality. Future studies may focus on sensorial behavior of radiated dried chillies produced in different countries.

## Data Availability

As this manuscript is submitted as a review article, data was from compilation of previously related studies, coming from various authors.

## Abbreviations

MPN: Most Probable Number; ETO, ethylene oxide; DNA, deoxyribonucleic acid; aw, water activity.
